# Reimagining Lignin Valorization: Synthetic Biology–Enabled Sustainable Aromatic Carbon Biomanufacturing

**DOI:** 10.1002/advs.76698

**Published:** 2026-07-20

**Authors:** Na Li, Jun‐Jie Zhangyang, Bing‐Zhi Li, Zhi‐Hua Liu, Ying‐Jin Yuan

**Affiliations:** ^1^ State Key Laboratory of Synthetic Biology Tianjin University Tianjin China; ^2^ School of Synthetic Biology and Biomanufacturing Tianjin University Tianjin China; ^3^ Frontiers Science Center for Synthetic Biology (Ministry of Education) Tianjin University Tianjin China; ^4^ The International Joint Institute of Tianjin University Tianjin University Fuzhou China

**Keywords:** lignin valorization, machine learning, metabolic engineering, microbial cell factories, synthetic biology

## Abstract

Lignin, the largest renewable aromatic carbon reservoir, represents a foundational yet underutilized feedstock for sustainable biomanufacturing. Despite decades of effort, its effective integration remains constrained, not only by inefficient depolymerization but critically by the lack of coordinated control across depolymerization, conversion, and metabolic regulation. Lignin's heterogeneity and dynamic derivative evolution undermine conventional pathway‐centric engineering, causing poor predictability and flux imbalances. This review proposes a paradigm shift from isolated catalytic steps toward an integrated depolymerization, conversion, and regulation framework, where synthetic biology provides the design logic to sense and manage lignin‐derived chemical complexity. Emerging technologies like photo‐enzymatic catalysis and chemo‐biological hybrids expand the design space for selective depolymerization. At the cellular level, microbial cell factories funnel heterogeneous aromatics into defined metabolic nodes. Crucially, these developments converge on a central insight: regulatory control, rather than pathway completeness alone, governs the efficiency, robustness, and scalability of lignin bioconversion. Global transcriptional regulation, dynamic biosensor‐based control, and growth–production decoupling establish systems‐level governance over carbon flux. By integrating dynamic regulation with modular pathways, lignin is transformed from an unpredictable substrate into a programmable aromatic feedstock. This work outlines a roadmap for lignin valorization, positioning synthetic biology‐enabled regulation as the unifying principle for sustainable aromatic carbon biomanufacturing.

## Graphical abstract

The diagram illustrates a sustainable model of lignin valorization and carbon cycling based on microbial cell factories. Using lignin as the feedstock, synthetic biology technologies, including depolymerization, cell factory construction, pathway gene module integration, and metabolic network regulation, are applied to convert lignin into bulk chemicals, natural products, and biodegradable materials within microbial cell factories.

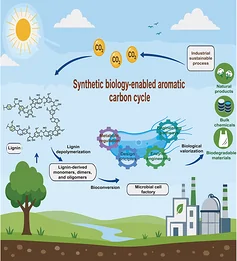

## 1 Introduction

Lignin, accounting for approximately 10%–35% of lignocellulosic biomass, represents the largest renewable reservoir of aromatic carbon in nature. Unlike carbohydrates, lignin is intrinsically enriched in functionalized aromatic structures, making it a unique and potentially sustainable platform for the production of fuels, chemicals, and advanced materials [1, 2]. Effective lignin valorization is therefore increasingly recognized as a key enabler for advancing biomass‐based circular bioeconomy [3, 4]. However, despite its abundance and chemical richness, lignin remains substantially underutilized, largely due to its structural complexity and resistance to selective depolymerization and biological conversion.

Structurally, lignin is a highly heterogeneous alkyl–aromatic polymer derived from three phenylpropanoid units‐syringyl (S), guaiacyl (G), and *p*‐hydroxyphenyl (H)‐that are interconnected through a diverse array of carbon‐carbon and carbon‐oxygen linkages [4]. This intrinsic heterogeneity, together with variable side‐chain functionalities and macromolecular features, yields depolymerization products that are difficult to predict and refractory to downstream bioconversion. These characteristics fundamentally challenge conventional chemical and biological upgrading strategies, underscoring the need for more adaptable and programmable valorization routes.

Physicochemical approaches, including catalytic depolymerization, pyrolysis, hydrogenolysis, oxidation, and solvent‐assisted fractionation, benefit from high reaction rates, robust process performance, and relative industrial maturity, enabling efficient lignin deconstruction and large‐scale operation [5, 6, 7]. Recent advances in catalyst design and lignin‐first biorefining have substantially improved aromatic monomer yields and process economics [8]. Nevertheless, lignin heterogeneity often results in poor product selectivity, complex product distributions, catalyst deactivation, and energy‐intensive processing under harsh reaction conditions [8, 9, 10]. Consequently, increasing attention has been directed toward biological lignin valorization that leverages enzyme specificity and microbial metabolism to achieve selective lignin upgrading under mild conditions while preserving aromatic functionalities.

Biological lignin valorization has emerged as a promising alternative to overcome these limitations by harnessing enzymatic catalysis and microbial metabolism. In natural systems, ligninolytic microorganisms employ extracellular oxidative enzymes, such as peroxidases and laccases, to initiate radical‐mediated lignin depolymerization. This process generates a complex mixture of lignin‐derived aromatic compounds (LDACs), such as dimers and monomers. These intermediates can subsequently be assimilated and transformed through diverse intracellular catabolic and anabolic pathways. Importantly, biological lignin conversion routes not only facilitate selective transformation under mild conditions but also preserve lignin's intrinsic aromaticity and functional group diversity [11].

Recent advances at the interface of biocatalysis and synthetic biology have opened new opportunities for lignin valorization [12]. The depolymerization of macromolecular lignin into membrane‐permeable oligomers or monomers constitutes the foundational initial step. Following depolymerization, the resulting bioavailable oligomers or monomers can be directed into three major biological routes depending on the desired product outcome. “Biological funnel” routes converge heterogeneous aromatics into central intermediates for bulk aromatic chemicals [13]. Atom‐economic functionalization routes preserve aromatic rings for the biosynthesis of high‐value aromatic natural products like flavonoids. While aromatic ring‐cleavage route yields a high‐value dicarboxylic acid. These routes collectively illustrate the metabolic versatility of biological systems for producing a broad spectrum of lignin‐derived products.

Despite this progress, several critical bottlenecks continue to constrain the efficiency and scalability of biological lignin conversion routes. The inherent recalcitrant C─C linkages limit depolymerization efficiency, while the chemical diversity and toxicity of LDACs impose significant metabolic stress on microbial hosts. Moreover, the construction of efficient and robust lignin‐converting cell factories is hindered by challenges in pathway assembly, flux balancing, and dynamic regulation across complex metabolic networks. Overcoming these barriers requires systematic reconstruction of synthetic biological routes, guided by design principles rather than empirical optimization alone.

**FIGURE 1:**
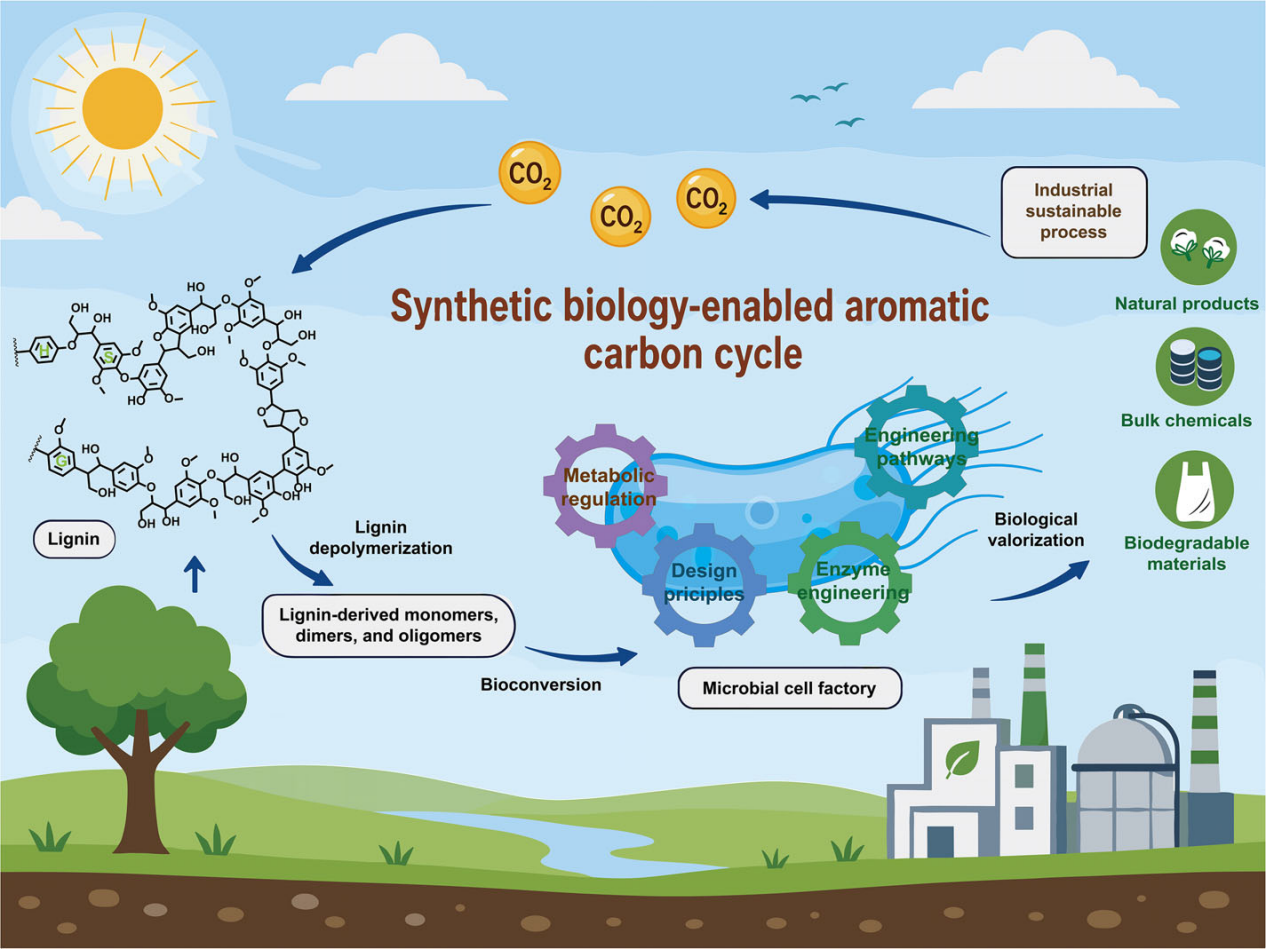
The diagram illustrates a sustainable model of lignin valorization and carbon cycling based on microbial cell factories. Using lignin as the feedstock, synthetic biology technologies, including depolymerization, cell factory construction, pathway gene module integration, and metabolic network regulation, are applied to convert lignin into bulk chemicals, natural products, and biodegradable materials using microbial cell factories.

This work aims to adopt a systems‐level perspective on synthetic biology‐enabled aromatic carbon biomanufacturing. We summarize the latest advances in reconstructing synthetic biology‐enabled lignin valorization routes, with a particular focus on strategies that integrate depolymerization, pathway engineering, and systems‐level regulation (Table 1). Emerging strategies, including photo‐enzymatic catalysis, synergistic multi‐enzyme assemblies, and integrated chemo‐biological processes, are highlighted as promising solutions to improve lignin depolymerization and substrate accessibility for downstream bioconversion. Advanced pathway engineering, modular route assembly, and metabolic network optimization were combined to enable efficient conversion of heterogeneous lignin streams. By integrating those technological frontiers, this review provides a conceptual framework for reconstructing robust and scalable lignin valorization routes, ultimately accelerating the transition toward a circular bioeconomy (Figure 1).

**TABLE 1:** Key strategies and cutting‐edge technologies for lignin valorization.

Stages	Key challenges	Strategies	Representative tools	Results/Performances	Ref.
Lignin depolymerization	High C─C bond energy Repolymerization of cleavage fragments Low yield of bioaccessible monomers	Photo‐enzymatic catalysis	Reductive photoenzymatic O‐demethylation	O‐Demethylation of aryl methyl ethers to methane	[105]
			A laccase‐methylene blue coupling system	Guaiacol degradation rate increased to 91.5%	[25]
			De novo metalloenzyme for cerium photocatalysis	Cleavage of lignin surrogates	[23]
			Lignin‐H_2_O_2_‐dependent UPO integration	Highly enantioselective C─H oxyfunctionalization	[18]
			A compartmented photo‐electro‐biochemical cell	98.7% selectivity for lignin depolymerization	[28]
		Synergistic multi‐enzyme complexes	Laccase‐LPMO‐ascorbic acid coupled system	40.9% alkali lignin depolymerization ratio Inhibition of lignin repolymerization	[36]
			De novo enzyme‐nanozyme hybrid system	Lignin degradation rate increased to 25.2%	[41]
			DypBs auxiliary complex system	Lignin utilization reached 25.0% 27.0% reduction in average molecular weight	[40]
			Synergistic LPMO‐ligninolytic enzymes	Driving the Fenton reaction for lignin degradation	[37]
			Synergistic action of laccase with LiP and MnP	Lignin degradation rate reached 25.8% Producing more acidic compounds	[33]
			A laccase‐Lig multienzymatic multistep system	Cleavage of non‐phenolic *β*‐O‐4 aryl ether bonds	[39]
			Combined quinone reductase and LiP	Inhibition of lignin repolymerization 31%–52% lignin molecular weight reduction	[35]
			LPMOs‐glucose dehydrogenase system	Enhanced Fenton reaction for lignin degradation	[106]
		Integrating the chemo‐biological process	Lignin fractionation‐laccase depolymerization	Highest PHA yield of 0.4 g/L	[44]
			Integrated γ‐valerolactone pretreatment, catalytic hydrogenolysis, and microbial funneling to PDC	Yield of 103 g PDC per kg lignin	[48]
			Pretreatment‐coupled multi‐enzyme deconstruction	Reduction of *β*‐O‐4 linkages by 10.5% and *β*‐*β* linkages by 50.0%	[46]
			An efficient enzyme–mediator–microbial system	Cell growth increase of 106‐fold Lipid titer reached 1.0 g/L	[107]
Microbial cell factory construction	Poor host metabolic capacity and robustness Chemical diversity of LDACs Intermediate accumulation and metabolic imbalance Cofactor limitation Inefficient expression and poor catalytic performance of key enzymes	Genome streamlining	Engineered *P. putida* KTU‐U13 and U27	Producing medium‐chain‐length PHA of 26.9 wt.% and 45.2 wt.%, respectively	[57, 58]
		Adaptive laboratory evolution	Adaptively evolved *P. monteilii* DM1‐E10	Producing 99 mg/L PHAs from kraft lignin	[59]
		Metabolic pathway modularization design	Modular engineering for adipic acid production	Record adipic acid production of 110 g/L in *E. coli*	[60]
		Precise control of metabolic flux	Rate‐limiting VanAB and PobA overexpression	A catechol titer of 8.8 mM	[61]
			CarR‐based *p*‐coumaric acid biosensor	FjTALG85S mutant with 6.9‐fold enhanced catalytic activity	[62]
		Cofactor regeneration	iMECS strategy	Coenzyme‐free synthesis of curcumin, etc. (> 90% conversion)	[65]
			EcdB overexpression for PrFMN regeneration	14.1 mM catechol titer at 98.5% molar yield	[61]
		Promoter engineering	Endogenous strong promoter screening	Record‐high PHA yield (1.7 g/L, 42 wt.%)	[69]
		Enzymes fusion	HpaB‐HpaC fusion with a (GGGGS)_3_ linker	Over 100% increase in protocatechuic acid yield	[55]
		Auxiliary protein co‐expression	Auxiliary protein co‐expression with protocatechuate decarboxylase	Muconic acid titer doubled to 4.9 g/L	[70]
		Enzyme design	Rational protein engineering of P450BM3	Enables guaiacol O‐demethylation with 100% selectivity	[71]
			GcoA‐F169A variant	Enables efficient syringol O‐demethylation	[72]
Synthetic biology tool development	Metabolic enzyme deficiency Low gene‐editing efficiency Difficult pathway assembly	Enzyme mining and discovery	RB‐TnSeq	Identification of genes essential for LDAC catabolism	[75]
			Direct cloning of BGCs	Efficient capture and expression of large gene clusters	[76]
			Multi‐omics guided pathway elucidation	Elucidation of novel *β‐β* lignin dimer catabolism	[77]
		Precision genome editing	Phage‐assisted PEHR‐Cas3 editing	High‐efficiency, markerless genome editing	[79]
			Donor‐free multiplex base editors	Enables single‐gene knockout and multiplex base editing	[80]
			GENIO platform for phenotype engineering	Optimization of heterologous pathway integration	[81]
		Large‐fragment pathway assembly	Modular in vitro assembly	Hierarchical assembly of large pathway constructs	[82, 83]
			In vivo direct assembly	In vivo DNA assembly in non‐model microorganisms	[85]
			I‐SceI‐mediated genomic integration	Stable integrated expression of assembled pathways	[84]
			Yeast life cycle‐assembly	Seamless assembly of pathways exceeding 100 kb	[82]
Global metabolic network regulation	Metabolic conflicts Cofactor imbalances Accumulation of toxic intermediates	Global dynamic regulatory	*Crc* knockout	44.5 g/L titer from model lignin compounds) and 25 g/L (corn stover streams)	[88, 89]
			Cofactor and energy balance	Quantitative metabolic coupling in *P. putida*	[90]
			Glucose uptake rate biosensors	Enhanced production of L‐tryptophan, etc.	[86]
		Genome‐scale metabolic models	Module‐predicted four‐gene deletions	Growth‐coupled glutamine production from *p*‐coumaric acid	[91]
			Manually GEM reconstruction	Predictive blueprint for lignocellulose fermentation	[92]
			GEM‐guided microbial consortia modeling	Optimized lignin degradation through synthetic consortia tuning	[93]
		Hybrid machine learning‐metabolic modeling framework	Enzyme‐constrained metabolic models	Enhanced predictive accuracy of genome‐scale models	[96]
			Cloud platforms such as CarveAdornCurate	Democratized modeling and accelerated design cycles	[97]
			ProEnsemble	3.7 g/L naringenin production via pathway balancing	[98]
			Integrated data processing protocols NOREVA	Optimal metabolomic data processing strategies	[99]
			Multi‐scale EM_iBsu1209‐ME framework	Advanced predictive understanding of cellular mechanisms	[100]

Abbreviations: APL, alkaline pretreated liquor; LiP, lignin peroxidase; MnP, manganese peroxidase; LPMOs, lytic polysaccharide monooxygenases; UPO, unspecific peroxygenases.

## 2 Emerging Technologies Addressing Lignin Depolymerization Bottlenecks

Efficient lignin depolymerization remains the primary bottleneck in biological lignin valorization, owing to the intrinsic heterogeneity and structural recalcitrance of this macromolecular aromatic polymer. While physicochemical strategies and native microbial enzymes can initiate breakdown, the selective cleavage of recalcitrant C─C linkages, suppression of repolymerization, and downstream separation of structurally similar monomers continue to limit practical deployment.

**FIGURE 2:**
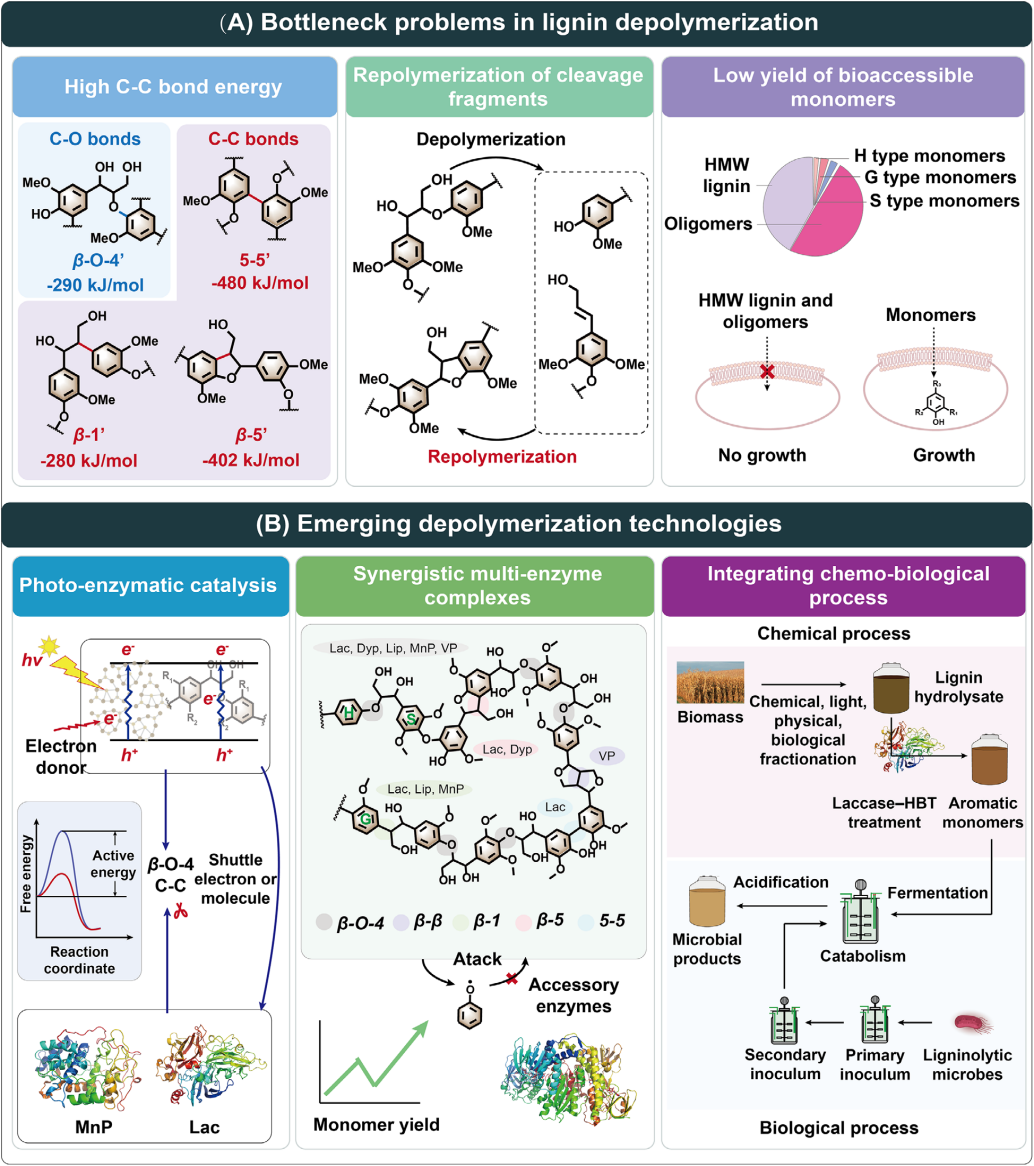
The major bottlenecks and emerging technological solutions in lignin depolymerization. (A) Key challenges include the high bond dissociation energy of C─C linkages in lignin, the repolymerization of cleavage intermediates, and the low yield of bioavailable monomers. Bond dissociation energies were calculated using density functional theory. (B) Three promising depolymerization strategies are highlighted: photo‐enzymatic catalysis, synergistic multi‐enzyme complexes, and integrated chemo‐biological processes, which aim to enhance the efficiency of lignin valorization.

To overcome these limitations, a new generation of depolymerization strategies has emerged that integrates principles from photo‐enzymatic catalysis, synergistic multi‐enzyme complexes, and integrated chemo‐biological processes (Figure 2) [14]. These approaches seek not only to improve bond cleavage efficiency but also to redirect reaction pathways toward biologically compatible intermediates.

### 2.1 Photo‐Enzymatic Catalysis: Expanding the Biocatalytic Design Space

Photo‐enzymatic catalysis has emerged as a promising strategy that couples the high reactivity of photocatalysis with the selectivity of enzymes [15, 16]. By harnessing visible light to generate redox equivalents or reactive oxygen species, photo‐assisted systems can overcome thermodynamic and kinetic constraints that often constrain the performance of natural ligninolytic enzymes [17].

Lignin itself has been shown to function as a photosensitizer, enabling light‐driven H_2_O_2_ generation that fuels oxidative biocatalysis [18]. Such findings highlight the possibility of developing self‐sustaining photo–bio hybrid systems that directly exploit lignin's intrinsic photochemical properties. For example, the H_2_O_2_‐dependent catalytic activity of unspecific peroxygenases (UPOs) and lytic polysaccharide monooxygenases (LPMOs) was enhanced to facilitate the enantioselective C─H oxyfunctionalization and oxidative cleavage of cellulose [18, 19]. The combination of reductive catalytic fractionation (RCF) pretreatment of lignin with photostimulated LPMO‐catalyzed enzymatic hydrolysis achieves a substantial 20% enhancement in the saccharification yield of lignocellulosic substrates under light‐driven conditions [20]. This insight opens avenues for developing specialized photo‐enzymatic systems that utilize light‐generated H_2_O_2_ to cleave the recalcitrant C─O/C─C bonds within the lignin macromolecule.

Beyond natural systems, synthetic photoenzymes constructed using gene engineering, chemical evolution, and immobilization strategies are beginning to expand the scope of enzymatic lignin depolymerization [21, 22]. Notably, de novo‐designed photoactive enzymes capable of visible‐light‐driven C─C bond cleavage exemplify how photoredox catalysis can be integrated into protein scaffolds to enable new‐to‐nature reactivity [23]. However, the initial variants of such de novo enzymes suffered from severe photodamage, which required multiple rounds of mutation to improve [23]. Similarly, different photoenzymatic systems have shown markedly different efficiencies. For example, direct electron transfer from [Ru(bpy)_3_]^2^^+^ to laccase is severely limited by inefficient energy transfer, and even with a methyl viologen relay, the yield reaches only ∼20% while photostability issues remain unresolved [24]. In contrast, a laccase–methylene blue system engineered with tight binding (7.3 Å) to the T1 Cu site bypasses FRET, achieving 91.5% guaiacol degradation and retaining >80% enzyme activity after 4 h, demonstrating that optimizing photosensitizer–active site geometry is more effective than relying on diffusible electron relays [25].

Nevertheless, incompatibility between photogenerated reactive species and enzyme stability remains a key challenge [15, 26]. Photogenerated holes and reactive oxygen species often inactivate enzymes, while the insulating nature of protein scaffolds often hinders efficient electron transfer. Emerging solutions, including spatial compartmentalization and membrane‐separated architectures, offer promising routes to protect biocatalysts while maintaining efficient charge or mediator transfer [27]. For example, a membrane‐ compartmentalized system integrating a TiO_2_ photocatalyst, a Co‐based electrocatalyst, and lignin peroxidase enables unassisted solar‐driven lignin valorization, achieving highly selective lignin dimer depolymerization (98.7% selectivity) and efficient biopolymer synthesis from coniferyl alcohol (73.3% yield) while preventing enzyme deactivation [28]. Furthermore, photo‐enzymatic catalysis is expanding from isolated enzymes to whole‐cell systems. A microbial photoelectrochemical platform in engineered *Escherichia coli* has been developed for light‐driven NADPH regeneration, demonstrating the potential of integrating solar energy conversion with cellular metabolism [29]. Collectively, these advances highlight the potential of photo‐enzymatic catalysis as a sustainable strategy for selective lignin depolymerization and integrated bioconversion.

### 2.2 Synergistic Multi‐Enzyme Complexes: From Single Catalysts to Functional Consortia

Natural lignin depolymerization rarely relies on individual enzymes, but instead arises from complex and synergistic enzymatic networks. Inspired by this principle, recent efforts have shifted from single‐enzyme systems toward engineered multi‐enzyme complexes capable of cooperative C─C and C─O bond cleavage and radical control [30].

Synergistic laccase‐peroxidase systems, for example, establish self‐sustaining catalytic cycles via in situ H_2_O_2_ generation, enhancing depolymerization efficiency by effectively cleaving critical chemical bonds of lignin, melanin, and pollutants while mitigating enzyme inactivation [31, 32]. The laccase‐lignin peroxidase‐manganese peroxidase complexes demonstrated approximately 29.0% degradation efficiency for alkaline lignin by destroying the macromolecular benzene ring structure of lignin and breaking the *β*‐O‐4, *β*‐5, 4‐O‐5, *β*‐1, and 5‐5 major bonds, thus producing more acidic compounds [33, 34].

A key advantage of multi‐enzyme systems lies in their ability to suppress lignin repolymerization, one of the major obstacles limiting monomer yields. Accessory enzymes such as quinone reductases, LPMOs, and radical‐scavenging oxidoreductases modulate redox balance, trap phenoxy radicals, and redirect reaction pathways away from undesired C─C coupling [35, 36, 37]. The flavin‐dependent dihydrolipoamide dehydrogenase from *Thermobifida fusca* was identified to prevent repolymerization by trapping phenoxy radicals [38]. Similarly, a laccase‐Lig multi‐enzyme cascade effectively depolymerized diverse lignin types, revealing that the phenolic/aliphatic hydroxyl ratio plays a critical role in governing repolymerization dynamics through its influence on radical coupling efficiency [39].

However, natural lignin depolymerization involves complex synergistic interactions among a broader enzymatic consortium rather than only two or three enzymes. Recent studies further demonstrate that rationally designed enzyme cocktails and enzyme–nanozyme hybrids can overcome steric constraints and amplify depolymerization efficiency beyond natural systems. For instance, a synthetic enzyme cocktail comprising 15 enzymes establishes quinone redox‐free radical catalytic networks that significantly amplify depolymerization efficiency [40]. But it alone degrades only approximately 8% of lignin, lacks activity on key OH groups, and its high cost and complex expression hinder scalability. The constructed λ‐MnO_2_ nanozyme‐laccase CotA hybrid system successfully overcomes the steric limitations of native multi‐enzyme systems, achieving 25.2% lignin depolymerization efficiency [41]. Laccase‐mediated methoxylation significantly enhanced the yield of aromatic compounds, but the nanozyme‐mediated ring‐opening activity was partially suppressed by laccase‐catalyzed methoxylation [41].

These findings suggest that lignin depolymerization should be viewed not as a single reaction, but as an emergent property of coordinated catalytic networks. The multi‐enzyme synergy strategy holds promise for cleaving recalcitrant bonds while minimizing repolymerization, thereby providing a green and sustainable solution for lignin valorization.

### 2.3 Integrating Chemo‐Biological Processes: Bridging Reactivity and Selectivity

While biological systems offer exceptional selectivity, chemical catalysis excels in breaking recalcitrant bonds. Integrating these complementary strengths has emerged as a powerful strategy for advancing lignin valorization. Chemo‐biological hybrid processes enable initial depolymerization or functionalization via chemical catalysis, followed by biological funneling or upgrading into single‐target products [42, 43].

In lignin valorization, integrated pretreatment, catalytic depolymerization, and microbial conversion have demonstrated improved monomer accessibility and enhanced bioconversion efficiency. For instance, combining lignin fractionation with laccase catalysis effectively reduces lignin molecular weight and enhances *β*‐O‐4 bond cleavage. Thereby, the bioconversion efficiency of lignin to polyhydroxyalkanoate was significantly improved with a titer of 0.4 g/L, which was 18% and 3.3% greater than those of untreated and laccase‐treated samples, respectively [44]. Similarly, laccase‐mediated oxidation of lignin following dilute acid pretreatment enhances lignin reactivity in formaldehyde‐based resin synthesis [45]. Leading pretreatment combined with mixed enzyme treatment has also been shown to reduce *β*‐O‐4 and *β*‐*β* linkages by 10.5% and 50.0%, respectively, thereby improving lignin reactivity during corn stover deconstruction [46].

The integration of enzymatic lignin depolymerization and electrocatalytic hydrodeoxygenation exhibits synergistic potential, enabling closed‐loop conversion of lignin into high‐value chemicals. However, advancing this strategy toward a circular bioeconomy will require breakthroughs in process compatibility and system‐level optimization [47]. Moreover, the consolidated bioprocessing (CBP) of lignin, which combines lignin depolymerization with microbial funneling bioconversion into single‐target products, represents a crucial route in biorefining [13]. Importantly, techno‐economic analyses indicate that combining chemical depolymerization with microbial funneling can significantly improve process viability. For example, integrating γ‐valerolactone pretreatment, catalytic hydrogenolysis, and microbial funneling achieved a yield of pyrone‐4,6‐dicarboxylic acid (PDC) up to 139 g/kg lignin, with a projected minimum selling price of $12.1/kg [48]. Another recent landmark study exemplifies the power of an integrated chemo‐biological redox process for lignin‐to‐adipic acid conversion. By integrating reductive catalytic fractionation, hydrodeoxygenation, Co/Mn/Br‐catalyzed autoxidation, and engineered *Pseudomonas putida*, lignin is converted to adipic acid at 26 wt.% yield, further demonstrating how chemo‑biological redox processes tackle C─C bond recalcitrance [49, 50]. Nevertheless, achieving seamless compatibility between chemical and biological stages remains a key challenge, particularly in terms of solvent tolerance, inhibitor management, and continuous operation. Addressing these issues will be critical for advancing integrated lignin biorefineries toward industrial relevance.

Overall, frontier technologies, including photo‐enzymatic catalysis, synergistic multi‐enzyme complexes, and integrated chemo‐biological processes, are redefining the conceptualization and implementation of lignin depolymerization. Rather than relying on single catalytic solutions, these approaches emphasize functional integration, system‐level coordination, and pathway reconstruction. By leveraging synergistic effects, these approaches demonstrate remarkable potential in cleaving recalcitrant C─C bonds, suppressing repolymerization, and improving monomer product yield, thereby collectively advancing lignin valorization toward a greener and more sustainable direction. However, factors such as enzyme production costs, catalyst stability, and reactor design continue to limit large‐scale industrial application. Compared with photo‐enzymatic catalysis and multi‐enzyme systems, integrated chemo‐biological processes may provide a promising route to balance efficiency and selectivity. Future progress will depend on improved mechanistic understanding, rational catalyst integration, and process‐level optimization of photo‐enzymatic catalysis and multi‐enzyme systems. Advancing these strategies will be essential for enabling precise lignin deconstruction and robust biological routes that support a circular biomass‐based economy.

## 3 Constructing Microbial Cell Factories for Lignin‐Derived Compound Conversion

**FIGURE 3:**
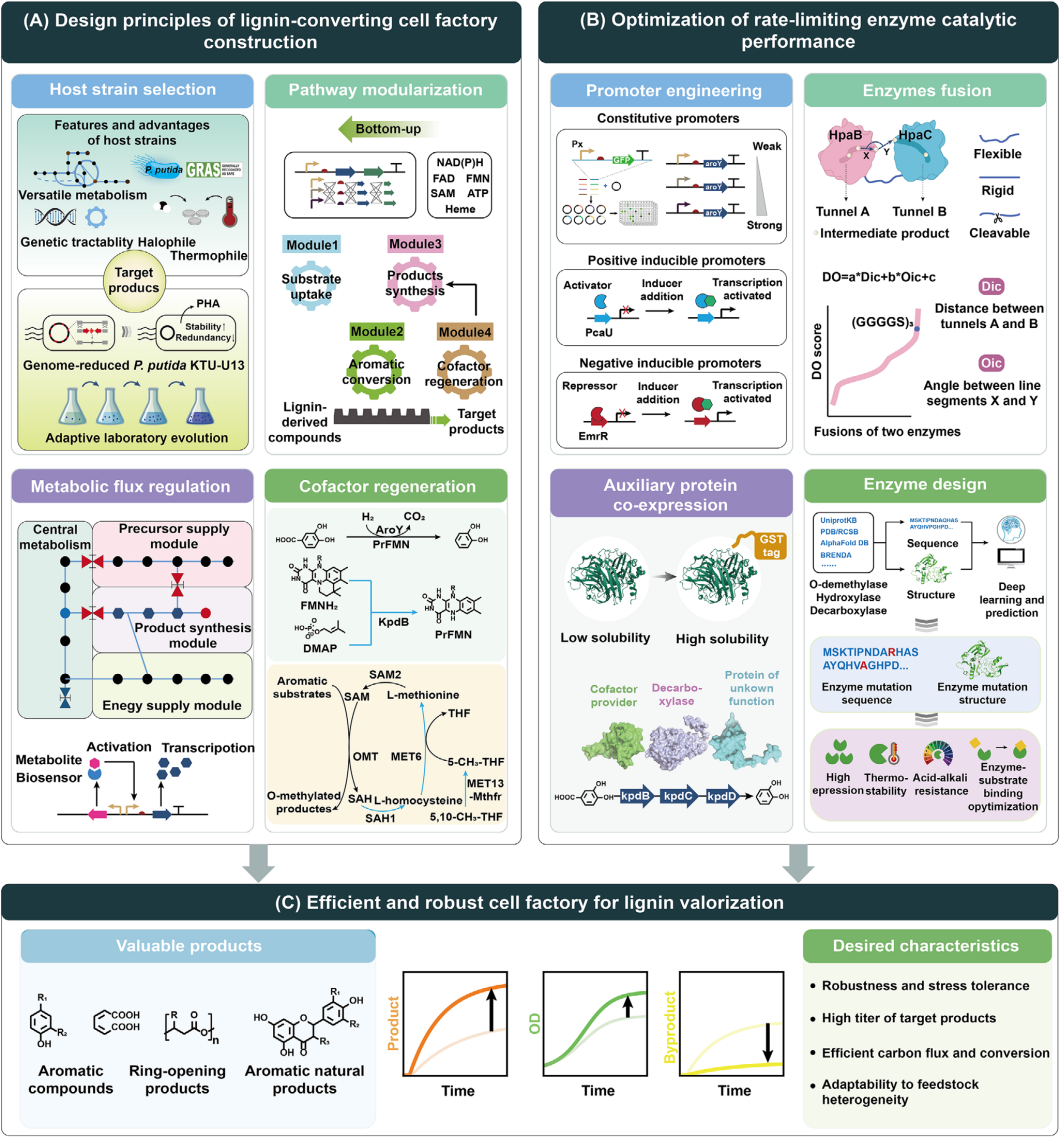
The design principles and enzyme engineering strategies for constructing microbial cell factories capable of lignin conversion. (A) The design principles cover host strain selection, pathway modularization, metabolic flux regulation, and cofactor regeneration. (B) Advanced enzyme engineering approaches include promoter engineering, enzyme fusion, and computational enzyme design. (C) The goal is to build efficient and robust cell factories capable of efficiently converting lignin into high‐value products, including aromatic compounds, ring‐opening derivatives, and aromatic natural products.

The construction of microbial cell factories capable of upgrading lignin‐derived compounds into high‐value products is a central pillar of biological lignin valorization. Following depolymerization, heterogeneous LDACs can be funneled through native or heterologous pathways into a limited set of platform compounds, such as protocatechuic acid, gallic acid, and catechol. However, inefficient expression and poor catalytic efficiency of key aromatic‐modifying enzymes, coupled with pathway incompleteness and aromatic toxicity, frequently constrain carbon flux and limit product titers. These challenges underscore the need for systematic design principles to reconstruct robust and efficient lignin‐converting cell factories (Figure 3).

Importantly, the performance of microbial cell factories is strongly influenced by the composition of lignin depolymerization streams. Different depolymerization technologies generate distinct distributions of aromatic monomers, dimers, oligomers, and inhibitory byproducts, which can substantially affect substrate uptake, cellular fitness, and metabolic flux allocation. Consequently, the selection and engineering of microbial hosts should be guided not only by their biosynthetic capabilities but also by their compatibility with the specific product spectrum generated during upstream lignin depolymerization.

The choice of an appropriate microbial chassis provides the foundation for lignin valorization. An ideal host should combine broad aromatic metabolic capacity, genetic manipulability, and environmental robustness. Different microbial chassis exhibit distinct advantages for lignin valorization. Despite their underdeveloped genetic tools, *Rhodococcus* species are increasingly explored for lignin biological valorization owing to their phenolic tolerance and lipid‐accumulating capability [51, 52, 53]. *Sphingobium* species are distinguished by their ability to cleave *β*‐aryl ether bonds and possess specialized pathways for aromatic compound degradation [54]. Engineered yeasts enable high‐value product synthesis via eukaryotic machinery, though their native aromatic pathways are weak and require extensive rewiring [55]. Among current candidates, *P. putida* KT2440 has emerged as a versatile and efficient candidate due to its native aromatic metabolism and exceptional robustness [54, 56]. Therefore, chassis selection should be matched to both the composition of depolymerization products and the target product portfolio. Genome streamlining and adaptive laboratory evolution have further enhanced host performance by improving robustness and metabolic efficiency. *P. putida* KTU‐U13 and U27, the genome‐streamlined strains, were successfully engineered with the capacity to produce medium‐chain‐length polyhydroxyalkanoate (PHA) at levels of 26.9% and 45.2%, respectively [57, 58]. Similarly, the adaptively evolved *P. monteilii* DM1‐E10 demonstrated bioconversion capability by producing 99 mg/L PHAs from kraft lignin compared to only 10.3 mg/L produced by the wild‑type strain, underscoring the importance of chassis optimization as a prerequisite for scalable lignin bioconversion [59].

Given the chemical diversity of LDACs, metabolic pathway modularization design has become a dominant strategy for reconstructing lignin conversion routes. By decomposing metabolism into functional modules, such as substrate uptake, intermediate conversion, product synthesis, and cofactor regeneration modules, each module can be individually optimized and subsequently integrated into a coherent production system. This bottom‐up strategy reduces system complexity, facilitates flux balancing, and enables rapid pathway reconfiguration in response to feedstock variability. For instance, modular engineering enabled record adipic acid production of 110 g/L from a mixture of cyclohexanol and cyclohexanone in *E. coli*, highlighting the broad applicability of modular design [60]. Under a cyclohexanol‐only feeding strategy, the engineered strain also produced approximately 22.6 g/L adipic acid, representing more than a two‐fold improvement over the previously reported titer of 10.2 g/L in *Pseudomonas taiwanensis* VLB120 [60]. By modularly constructing conversion pathways for lignin‐, petroleum‐, and plastic‐derived aromatic compounds, a biological funnel pathway for catechol synthesis was established, achieving a record titer of 14.1 mM and a molar yield of 98.5% from lignin derivatives [61]. Modularization thus provides a powerful framework for translating heterogeneous lignin streams into single‐target products.

Precise control of metabolic flux is essential to avoid intermediate accumulation and metabolic imbalance during lignin conversion. Transcriptional regulation, enzyme overexpression, and biosensor‐based feedback control have emerged as effective tools to dynamically tune pathway activity. In particular, genetically encoded aromatic‐responsive biosensors enable real‐time monitoring of intracellular metabolite levels, supporting both high‐throughput strain optimization and adaptive flux regulation. For example, overexpressing the rate‐limiting enzymes of VanAB and PobA minimized the accumulation of intermediates, such as vanillic acid and *p*‐hydroxybenzoic acid, allowing strain KTCA07 to produce 8.8 mM catechol with a molar yield of 96.7%. Relative to the parental strain KTCA02, catechol titer and yield were improved by 166.7% and 196.6%, respectively [61]. A *p*‐coumaric acid biosensor engineered from the CarR transcription factor enables high‐throughput screening through fluorescence‐activated cell sorting, facilitating rapid selection of an improved FjTAL^G85S^ mutant with 6.9‐fold enhanced catalytic activity [62]. These dynamic control strategies are increasingly recognized as critical components of next‐generation lignin cell factories.

Cofactor availability and regeneration exert a profound influence on lignin bioconversion efficiency, as many aromatic‐modifying enzymes depend on SAM, NAD(P)H, or specialized flavin cofactors. For example, a cofactor regeneration strategy targeting the unique prenylated flavin mononucleotide (PrFMN) requirement of protocatechuate decarboxylases enhanced catalytic activity, achieving a catechol titer of 14.1 mM with a molar yield of 98.5% [61]. Similarly, engineered SAM cycles can promote the supply and regeneration of methyl donors, thereby enhancing methylation conversion efficiency [63, 64]. The in vitro multienzyme‐coordinated expression with cofactor self‐circulation (iMECS) strategy represents a notable example of integrating modular enzyme assembly with cofactor engineering [65]. These studies demonstrate that targeted cofactor engineering can effectively alleviate redox and methyl‐donor limitations. More importantly, coordinating enzyme expression with cofactor self‐circulation provides a systems‐level strategy for enhancing pathway performance beyond enzyme‐centric approaches. In addition, cofactor engineering is closely linked to formaldehyde metabolism during lignin valorization. The O‐demethylation of methoxylated LDACs frequently generates formaldehyde, a toxic intermediate that can impair cell fitness and carbon utilization efficiency. Recent studies have shown that enhancing formaldehyde detoxification or assimilation pathways not only alleviates toxicity but also improves intracellular redox balance through the generation of NAD(P)H during formaldehyde oxidation [66]. Furthermore, the introduction of formaldehyde‐assimilating routes such as the ribulose monophosphate (RuMP) pathway can recycle one‐carbon units into central metabolism, thereby increasing carbon efficiency and supporting biomass formation [67]. These findings highlight formaldehyde metabolism engineering as an emerging complement to conventional cofactor engineering, simultaneously addressing redox homeostasis, carbon conservation, and stress tolerance in lignin‐converting microbial cell factories.

Beyond pathway architecture, the catalytic efficiency of rate‐limiting enzymes remains a major determinant of cell factory performance [68]. Recent advances in promoter engineering, protein fusion, auxiliary protein co‐expression, and structure‐guided enzyme design have enabled precise enhancement of enzyme activity, stability, and substrate specificity. For instance, screening endogenous strong promoters and chromosomally integrating the native P46 promoter upstream of key genes resulted in a record‐high PHA production, reaching an absolute titer of 1.7 g/L and a relative yield of 42 wt.%, representing increases of 165% and 90%, respectively, compared with the starting strain KTU [69]. In vanillin biosynthesis from lignin‐derived monomers, fusion of the hydroxylase HpaB and reductase HpaC via a (GGGGS)_3_ flexible linker alleviated rate‐limiting hydroxylation and methylation [55]. Similarly, co‐expression of an auxiliary protein enhanced protocatechuate decarboxylase activity, successfully overcoming the metabolic bottleneck in muconic acid biosynthesis by *P. putida*, achieving a more than doubled titer of 4.9 g/L [70]. Engineering of O‐demethylases currently represents a major focus in the modification of key enzymes within lignin metabolic pathways. Rational design of P450BM3 generated the V78A/F87A/T268I/A264G mutant, which achieved highly efficient O‐demethylation of guaiacol with 100% selectivity while circumventing the limitations of traditional NADPH‐dependent systems [71]. Likewise, the GcoA‐F169A variant overcomes steric hindrance to enable efficient syringol O‐demethylation, establishing the first microbial pathway for sinapyl alcohol‐derived lignin valorization [72]. Structure‐guided engineering of the P450 O‐demethylase AgcA further expanded substrate specificity toward both guaiacyl‐ and syringyl‐derived monomers, enabling efficient conversion of 4‐propylsyringol in an engineered *Rhodococcus* biocatalyst [73]. A DBTL framework incorporating quantum mechanics has been proposed for the rational design of phosphatase mutants [74]. Collectively, these approaches improve individual reaction steps, reduce metabolic burden, and improve pathway robustness, thereby expanding the operational window of lignin valorization routes.

Taken together, the construction of microbial cell factories for lignin valorization requires coordinated optimization across multiple biological scales, from chassis selection and pathway modularization to dynamic regulation, cofactor management, and enzyme engineering. Rather than focusing on incremental improvements of individual components, future progress will depend on the holistic reconstruction of synthetic biological routes that are adaptable to lignin heterogeneity and resilient under industrial conditions. These synergistic efforts are extending the frontiers of lignin valorization, paving the way for robust and efficient next‐generation microbial cell factories that support sustainable biomanufacturing from renewable lignin.

## 4 Engineering and Assembly of Complex Pathways for Lignin Valorization

**FIGURE 4:**
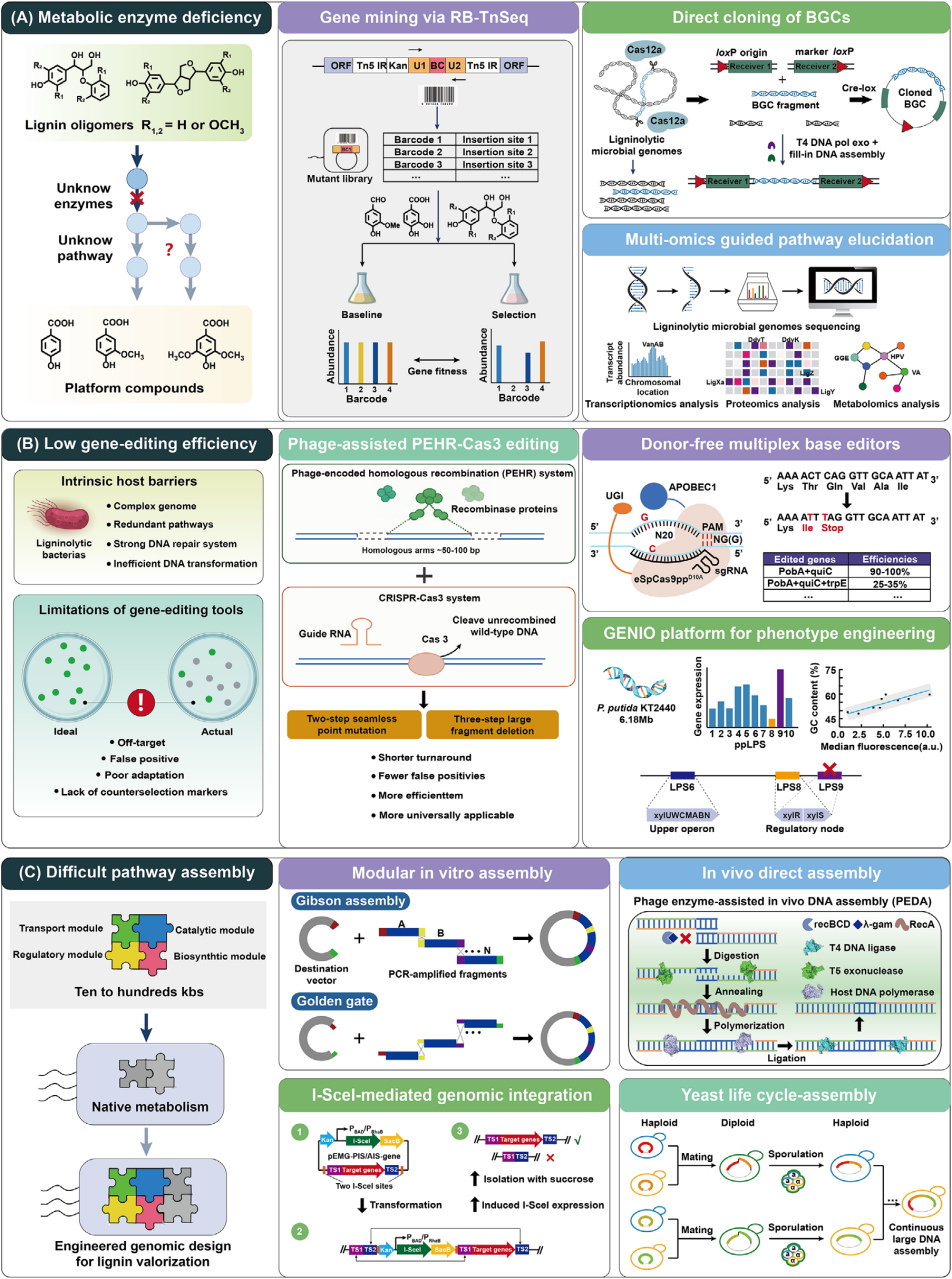
Key challenges and major technological advances in pathway construction and assembly for lignin valorization. The pathway construction and assembly for lignin valorization face several critical challenges, including metabolic enzyme deficiency, low gene‐editing efficiency, and difficulties in pathway assembly. (A) The mining of potential genes can be facilitated by methods such as RB‐TnSeq‐based gene mining, direct cloning of biosynthetic gene clusters (BGCs), and multi‑omics‑guided pathway elucidation. Created by the authors based on information reported in Ref. [75] and adapted from Ref. [76] under the Creative Commons Attribution 4.0 International License. (B) Advanced gene‑editing tools, including phage‑assisted PEHR‑Cas3 editing and donor‑free multiplex base editors, have been developed to enhance editing efficiency. Created by the authors based on information reported in Ref. [79, 80, 81]. (C) Furthermore, modular in vitro and in vivo pathway assembly techniques are employed to streamline the construction of complex microbial cell factories capable of lignin utilization across the entire process. Created by the authors based on information reported in Ref. [82, 84] and adapted with permission from Ref. [85]. Copyright 2022, American Chemical Society.

Lignin depolymerization generates highly heterogeneous LDAC mixtures, including monomers, dimers, and oligomers, which collectively challenge microbial assimilation and pathway integration. Many engineered hosts lack native pathways to efficiently process such chemically diverse substrates, while key heterologous enzymes essential for complete lignin valorization are often absent or poorly expressed. Furthermore, lignin metabolism is intrinsically polygenic and synergistic, necessitating coordinated manipulation of multiple genes and large genomic regions. This task exceeds the capacity of conventional genetic engineering approaches, particularly in non‐model organisms (Figure 4). Therefore, it is urgently necessary to promote the adoption of synthetic biology toolkits to tackle the inherent complexity of lignin metabolism and enable programmable aromatic compound bioproduction.

Beyond host selection, the composition of depolymerized lignin streams also shapes pathway assembly strategies. Monomer‐enriched streams enable streamlined funneling pathways, whereas mixtures containing diverse aromatics, dimers, or inhibitors require expanded catabolic networks, detoxification modules, and dynamic regulatory circuits. Therefore, effective pathway engineering should be considered in the context of the upstream depolymerization platform to maximize carbon utilization and product formation.

Lignin demethylases and dimer‐metabolizing enzymes from bacterial sources remain largely unexplored and await further development. Recent advances in enzyme mining have substantially broadened the repertoire of lignin‐converting biocatalysts. High‐throughput genetic screening tools, such as genome‐wide RB‐TnSeq (Random Barcode Transposon Sequencing) fitness profiling, enable the systematic identification of genes essential for LDAC catabolism within complex metabolic networks in *Sphingobium sp*. SYK‐6 [75]. Direct cloning techniques like CAPTURE (Cas12a‐assisted precise targeted cloning using in vivo Cre‐lox recombination) overcome cultivation limitations to efficiently capture and express large, complex biosynthetic gene clusters encoding lignin‐degrading enzymes [76]. When integrated with multi‐omics approaches including genomics, transcriptomics, and metabolomics, these approaches enable rapid elucidation of previously uncharacterized lignin catabolic pathways. For example, a novel bacterial pathway for *β*‐*β* lignin dimer (+)‐pinoresinol catabolism was elucidated in *Novosphingobium rhizosphaerae* LY [77]. Over twenty potential ligninolytic enzymes and nine catabolic pathways were identified in the fungus *Myrothecium wuxin* [78]. However, most of these predicted enzymes and pathways lack biochemical validation and require further experimental support to confirm their actual involvement in lignin degradation [78]. Overall, the convergence of functional genomics, advanced cloning, and multi‐omics provides a robust methodological foundation for expanding the biocatalytic toolbox available for engineering microbial chassis capable of transforming heterogeneous lignin streams.

Translating newly discovered enzymes into functional cell factories requires robust and precise genome engineering platforms. Several sophisticated, species‐specific toolkits have moved beyond reliance on a few model organisms toward the rational engineering of native ligninolytic microbes. For example, highly efficient and versatile genome‐editing toolkits have drastically accelerated design‐build‐test‐learn cycles for bacterial hosts of *Pseudomonas*. Beyond conventional CRISPR‐Cas9, novel systems such as the PEHR–Cas3 integrate phage‐encoded recombination with Cas3 nuclease activity, enabling efficient, markerless genome editing within approximately 12 days [79]. Furthermore, donor DNA‐free cytosine base editors enable not only single‐gene inactivation but also multiplex base editing, thereby accelerating the optimization of the lignin valorization pathway in *Pseudomonas* species [80]. However, their application is limited by sequence‐context preferences, stringent PAM requirements, and reduced efficiency during multiplex editing, which decreases to 25%–35% when three loci are targeted simultaneously [80]. Successful engineering of complex phenotypes requires not only efficient genetic tools but also deep integration of heterologous pathways into the host's native metabolic and genetic networks. Integrated platforms like GENIO systematically optimize heterologous pathway integration and host fitness by coupling rational locus characterization with adaptive laboratory evolution [81]. Overall, the field is rapidly moving beyond reliance on a limited set of model hosts toward the rational engineering of native lignin degraders. Precision genome engineering is emerging as a cornerstone for expanding the chassis landscape of lignin valorization.

Lignin valorization often requires the introduction of extensive heterologous pathways spanning tens to hundreds of kilobases and encompassing both catalytic and regulatory elements. Recent advances in large‐fragment DNA assembly technology have enabled modular construction and hierarchical integration of such pathways. Several highly efficient Golden Gate or Gibson assembly techniques have facilitated the construction of initial pathway segments (< 20 kb) and their integration into larger constructs via the yeast life cycle‐assembly method or HAnDy, enabling seamless assembly of pathways exceeding 100 kb [82, 83]. The assembled pathways are then stably integrated into the genome using precision tools such as I‐SceI‐mediated systems, ensuring controlled expression and stability [84]. However, integration efficiency is often site‐dependent, resulting in variable outcomes across genomic loci. Notably, in vivo DNA assembly strategies further bypass the limitations of traditional cloning workflows, enabling pathway construction directly within non‐model microorganisms. The “phage enzyme‐assisted in vivo DNA assembly” (PEDA) method, which employs combinatorial expression of T5 exonuclease and T4 DNA ligase, facilitates in vivo assembly of DNA fragments in multiple microorganisms [85]. However, their efficiency is host‐ and RecA‐dependent, declines with increasing fragment numbers, and remains constrained in complex multi‐fragment assemblies [85]. These capabilities represent a critical step toward programmable, genome‐scale reconstruction of lignin conversion pathways.

Collectively, the convergence of enzyme mining, precision genome editing, and large‐fragment pathway assembly is transforming the engineering of complex lignin valorization routes. Rather than relying on incremental pathway extension, the field is shifting toward holistic reconstruction of metabolic capabilities at the genome scale. However, remaining challenges, including incomplete understanding of lignin oligomer conversion pathways, metabolic burden imposed by large heterologous pathways, and the lack of universal genetic toolkits, underscore the continued need for methodological innovation. Addressing these gaps will be essential to enable efficient biological upgrading of heterogeneous lignin depolymerization streams.

## 5 Global Metabolic Network Regulation in Lignin Biovalorization

**FIGURE 5:**
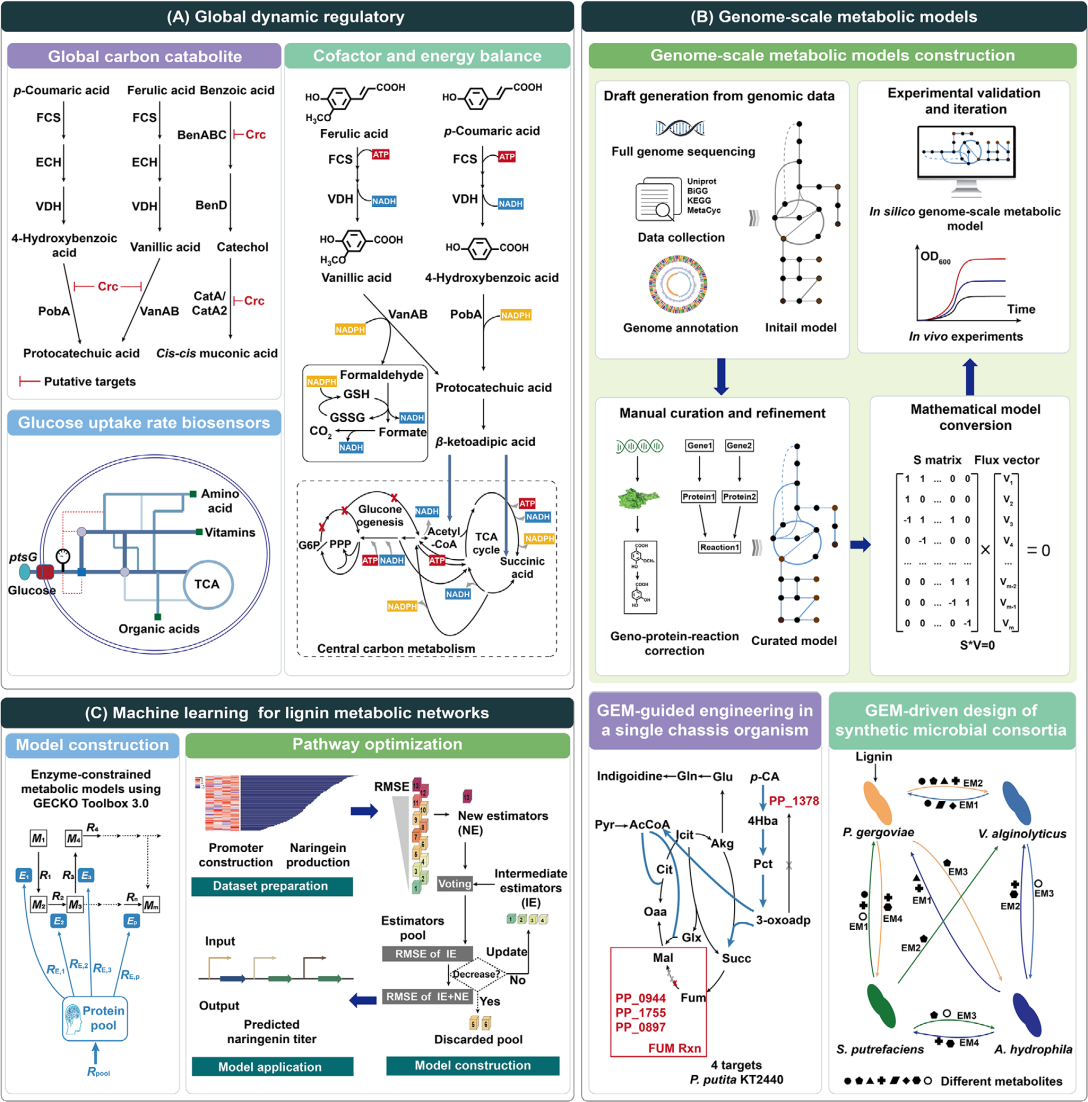
Global dynamic regulation strategies for optimizing lignin metabolic networks, genome‐scale regulation models, and machine learning‐assisted lignin regulatory networks. (A) Global carbon catabolite regulation, cofactor and energy balance, and substrate uptake rate biosensors help dynamic regulation of metabolic networks. Created by the authors based on information reported in Ref. [86, 89, 90]. (B) The development of GEMs typically involves four key steps: draft reconstruction, manual curation and refinement, mathematical model conversion, and experimental validation and iteration. These tools enable the rational design of both single‐chassis organisms and synthetic microbial consortia, thereby enhancing lignin conversion efficiency. Created by the authors based on information reported in Ref. [91, 93]. (C) Machine‐learning approaches facilitate the design and regulation of lignin's metabolic networks by supporting pathway prediction, enzyme engineering, and genome‐scale metabolic modeling. In the GECKO enzyme‐constrained metabolic model, M represents metabolites, R denotes chemical reactions, and E signifies enzyme components. Created by the authors based on information reported in Ref. [96] and adapted from Ref. [98] under the Creative Commons Attribution 4.0 International License.

Ligninolytic microbes operate through highly interconnected, complex aromatic metabolic networks that coordinate substrate assimilation, redox balance, and energy generation. Traditional metabolic engineering strategies, which typically focus on individual genes or localized pathways, are often insufficient for managing such system‐level complexity. In lignin valorization, this limitation manifests as metabolic conflicts, cofactor imbalances, and accumulation of toxic intermediates, ultimately constraining strain robustness and productivity. These challenges necessitate a shift from local pathway optimization toward global metabolic network regulation (Figure 5).

Efficient conversion of heterogeneous lignin mixtures necessitates global metabolic regulation of central carbon metabolism, energy balance, and cofactor homeostasis. The ability to perceive and respond to global metabolic signals, such as substrate uptake, enables real‐time adjustment of metabolic states in response to changing intracellular and extracellular conditions. For example, programmable glucose uptake rate biosensors dynamically monitor and regulate central carbon metabolism, allowing fine‐tuning of this pathway to optimize biosynthesis [86]. A dynamic two‐stage strategy that utilizes a nitrogen starvation‐responsive biosensor to decouple growth from production enables efficient accumulation of itaconic acid [87]. However, the response range and stability of the sensors often limit their versatility. A critical barrier in lignin valorization is hierarchical carbon catabolite repression (CCR), which prioritizes sugars over aromatics and hinders mixed substrate co‐utilization. Targeted intervention in CCR mechanisms, such as disrupting the catabolite repression control global regulator *crc* or employing CRISPRi‐mediated *crc* knockdown, has proven effective in enhancing co‑utilization efficiency and aromatic catabolism [88]. For example, *crc* knockout in an engineered *β*‐ketoadipate‐producing strain enhanced aromatic uptake and redirected carbon flux, achieving titers of 44.5 g/L from model lignin‐related compounds and 25 g/L from corn‐stover‐derived streams [89]. Moreover, growth on aromatic substrates induces extensive metabolic rewiring, activating auxiliary pathways that support redox and energy demands. In *P. putida*, growth on phenolic substrates activates routes such as pyruvate carboxylase and the glyoxylate shunt. It not only sustains carbon flow but also efficiently generates key cofactors (NADH/NADPH) and two‐fold higher ATP, addressing the critical redox and energy demands of biosynthesis [90]. Importantly, substrate structure and composition dictate metabolic rewiring, with 2.5 to 13‐fold higher glyoxylate shunt flux for hydroxycinnamates than hydroxybenzoates and a six‐fold greater NADH than NADPH surplus, rendering NADPH‐dependent enzyme engineering more vulnerable to cofactor deficiency [90]. Overall, harnessing these global responses represents a powerful lever for improving lignin bioconversion efficiency.

Genome‐scale metabolic models (GEMs) provide computational platforms for rationally navigating the complexity of lignin metabolism. By integrating known biochemical reactions into a computable framework, GEMs enable prediction of metabolic bottlenecks, identification of genetic intervention targets, and evaluation of pathway–host compatibility. GEM‐based simulations have enabled precise designs in key chassis microorganisms. For example, module‐predicted four‐gene deletions in *P. putida* KT2440 successfully rewired central metabolism to couple growth with glutamine production from *p*‐coumarate [91]. Similarly, a manually curated GEM for *Ruminiclostridium cellulolyticum* H10 has provided a predictive blueprint for simulating and subsequently enhancing lignocellulose fermentation pathways in related organisms [92]. Beyond single‐organism engineering, GEMs have generated fundamental insights into synthetic microbial consortia, revealing how precise metabolite cross‐feeding and population structure shape lignin degradation efficiency, with functional optimization depending on optimized subpopulation ratios [93]. Nevertheless, the predictive performance of GEMs varies substantially across organisms and environmental conditions. Many models rely on incomplete reaction annotations, simplified biomass equations, and steady‐state assumptions that may not accurately capture the dynamic and heterogeneous nature of lignin‐derived substrate utilization. Consequently, predictions that appear optimal in silico do not always translate into the expected phenotypes in vivo. One illustrative example is the predicted four‐gene deletion design, which did not yield the expected high glutamine production because fumarase hydratase unexpectedly emerged as the new rate‐limiting step [91]. Overall, GEMs are powerful digital tools for guiding metabolic engineering in lignin valorization. Continued expansion of multi‐omics datasets, coupled with rigorous manual curation and advanced modeling toolkits, will accelerate the development of high‐fidelity models and broaden their practical application.

Machine learning is increasingly reshaping the design and regulation of biological lignin metabolic networks by enabling data‐driven prediction and optimization across multiple biological scales [94, 95]. Integration of machine learning with metabolic modeling enhances parameter estimation, improves kinetic realism, and accelerates pathway balancing in microbial cell factories for lignin conversion. For example, GECKO 3.0 integrates deep learning‐predicted enzyme kinetics and proteomics data to construct enzyme‐constrained metabolic models (ecModels), significantly enhancing the predictive accuracy of genome‐scale models [96]. Cloud‐based platforms like CarveAdornCurate democratize advanced modeling by providing accessible tools, thereby reducing dependence on manually curated networks and accelerating the rational design cycle for non‐specialists [97]. In parallel, machine learning–guided optimization of gene expression and enzyme ensembles enables systematic debottlenecking of complex pathways. ProEnsemble can balance the biosynthesis pathway by optimizing the transcription of individual genes, achieving 3.7 g/L naringenin production in engineered *E. coli* [98]. Furthermore, machine learning revolutionizes data processing and multi‐scale analysis. Integrated protocols such as NOREVA systematically evaluate thousands of combinatorial data processing workflows through multi‐criteria assessment to determine optimal processing strategies for specific metabolomic studies [99]. Moreover, the multi‐scale EM_iBsu1209‐ME framework integrates machine learning with metabolic networks to enable system‐level analysis of cellular processes across scales. This framework is validated using an extensive normalized *Bacillus subtilis* compendium encompassing gene expression and metabolite synthesis data, thereby advancing predictive understanding of complex cellular mechanisms [100]. Despite these advances, several challenges remain for AI‐guided lignin valorization. The performance of machine learning models is often limited by the scarcity, heterogeneity, and lack of standardization of lignin‐related biological datasets [101]. Additionally, many advanced algorithms suffer from limited interpretability, making it difficult to elucidate the biological basis of their predictions and guide experimental validation [102]. Improving data quality, model transparency, and integration with mechanistic biological knowledge will be critical for the broader adoption of AI‐assisted biological design [103]. Overall, these approaches pave the way toward intelligent and adaptive microbial cell factories that can respond to lignin heterogeneity.

Global metabolic network optimization, including dynamic regulation, genome‐scale modeling, and machine learning, marks a decisive transition from reductionist engineering toward systems‐level control in lignin valorization. Despite remaining challenges in model predictability, pathway–host coordination, and strain robustness, the ongoing convergence of synthetic biology, systems engineering, and artificial intelligence is expected to unlock new levels of efficiency and scalability. Ultimately, these advances will enable intelligent microbial platforms for the sustainable and economically viable bioprocessing of lignin.

## 6 Concluding Remarks and Future Perspectives

Biological lignin valorization has transitioned from the exploration of native catabolic pathways toward the deliberate reconstruction of synthetic biological routes. This shift reflects a broader paradigm in which lignin is no longer viewed as a problematic byproduct but as a programmable aromatic feedstock. By integrating synthetic biology with advanced biocatalysis, pathway engineering, genome editing, and systems‐level regulation, increasingly sophisticated strategies are emerging to address long‐standing challenges associated with lignin heterogeneity, C─C bond recalcitrance, and metabolic complexity.

Despite this progress, reconstructing efficient and scalable biological lignin valorization routes remains nontrivial. Incompatibilities among catalytic components, such as mismatched operational windows between photo‐, chemo‐, and biocatalysts, continue to limit system integration [104]. Moreover, many promising biological technologies remain at the proof‐of‐concept stage, with insufficient validation under industrially relevant conditions. Overcoming these limitations will require not only technological refinement but also a deeper understanding of catalyst connectivity, pathway robustness, and process‐level compatibility. Importantly, a substantial gap still exists between laboratory‐scale demonstrations and commercially viable deployment. Comprehensive techno‐economic assessments under realistic operating conditions remain scarce, limiting the ability to accurately evaluate the industrial competitiveness of emerging biological routes. Beyond biological and engineering challenges, industrial translation faces practical barriers that widen the gap between laboratory demonstrations and realistic alternatives. Process integration across depolymerization, conversion, and product recovery stages is hindered by mismatched operating conditions, catalyst compatibility, and process stability. The inherent variability of lignin feedstocks arising from biomass source and pretreatment complicates standardization and reduces product yield consistency. Downstream separation also represents major economic bottlenecks, especially for low‐concentration products in complex aromatic mixtures. Addressing these challenges requires the development of robust feedstock‐adaptive platforms, integrated biorefinery strategies, and cost‐effective separation technologies capable of supporting large‐scale implementation.

Given these practical constraints, biological lignin valorization must be evaluated in the broader context of established physicochemical technologies. Biological routes currently exhibit low technology readiness and often suffer from slow conversion rates, low productivity, and great sensitivity to substrate variability. Consequently, biological processes are not yet positioned to replace physicochemical approaches for large‐scale bulk chemical production. However, biological systems offer unique advantages in substrate funneling, product selectivity, and functional‐group preservation. In particular, the ability to convert heterogeneous lignin‐derived aromatic mixtures into a single target molecule may reduce downstream separation requirements and improve overall process efficiency. Therefore, the most promising future scenario may not be competition between biological and physicochemical routes, but their integration within hybrid biorefinery frameworks.

Looking ahead, the convergence of synthetic biology, systems engineering, and machine learning is poised to redefine lignin valorization. Data‐driven design, coupled with dynamic regulation and autonomous optimization, will enable predictive reconstruction of lignin conversion routes that are adaptive to feedstock variability and resilient to metabolic perturbations. Ultimately, advancing from modular demonstrations toward integrated, self‐optimizing bioprocesses will be essential for translating lignin valorization from laboratory innovation into an enabling technology for hybrid biorefineries and a cornerstone of the emerging circular bioeconomy.

## Author Contributions

**Na Li**: writing – original draft, conceptualization, visualization, writing – review and editing. **Jun‐Jie Zhangyang**: writing – review and editing, visualization. **Bing‐Zhi Li**: resources, writing – review and editing, funding acquisition. **Zhi‐Hua Liu**: conceptualization, visualization, funding acquisition, supervision, writing – review and editing, resources. **Ying‐Jin Yuan**: resources, funding acquisition.

## Conflicts of Interest

The authors declare no conflicts of interest.

## Data Availability

The data that support the findings of this study are available from the corresponding author upon reasonable request.
